# Barriers and potential solutions for improved surgical care for children with hernia in Eastern Uganda

**DOI:** 10.1038/s41598-021-90717-2

**Published:** 2021-05-31

**Authors:** Mary Margaret Ajiko, Jenny Löfgren, Solvig Ekblad

**Affiliations:** 1 Soroti Regional Referral Hospital, Soroti, Uganda; 2 Department of Molecular Medicine and Surgery, Karolinska Institutet, Karolinska University Hospital, L1:00, 17176 Stockholm, Sweden; 3 Department of Learning, Informatics, Management and Ethics (LIME), Cultural Medicine, Karolinska Institutet, Stockholm, Sweden

**Keywords:** Paediatric research, Health care, Health services, Public health

## Abstract

Five billion people lack timely, affordable access to surgery. A large proportion of these are children. Qualitative research investigating the barriers to surgical care for children and ways of overcoming them is lacking. This study focused on children with hernia, a very common paediatric surgical condition for which surgery is the only effective treatment. The main aim of this qualitative study was to explore barriers to surgical care for children and identify potential solutions. Data were collected from parents of children with hernia and from health care providers at Soroti Regional Referral Hospital in eastern Uganda. Parents’ experiences, motives and barriers when accessing care were explored. The health care providers’ knowledge, perceptions and practices relating to children with hernia were investigated. The data were analysed using thematic content analysis. Traditional beliefs and gender inequality were considered major issues. Possible solutions included partnering with the local community in efforts to increase knowledge and acceptability in the community in general and by parents in particular. A formation of a surgical team dedicated to the management of children with surgical conditions was suggested as way to improve quality and increase volume of surgery for children.

## Introduction

Child health has received particular attention both in the United Nations’ Millennium Development Goals and the subsequent Sustainable Development Goals as part of Agenda 2030. Great progress has been achieved and the global under-five mortality rate declined by 59%, from 93 deaths per 1000 live births in 1990 to 38 in 2019. Still, an estimated 5.2 million children under the age of five died in 2019^1^. Sustainable Development Goal 3 sets out to “ensure healthy lives and promote well-being for all at all ages.” Target 3.2 relates specifically to child health and sets out to “end preventable deaths of new-borns and children under 5 years of age.”^2^. Sub-Saharan Africa has the world’s highest under-five mortality rate and half of the global under-5 deaths occur here^3^. To achieve the ambitious health goals, children’s access to surgical services of high quality must be ensured.

Each year, 303,000 children die due to congenital anomalies and even more from trauma^4,5^. Surgery could save many of these deaths and in addition would reduce morbidity. Surgical conditions in children are prevalent in low- and middle-income countries but many never seek or receive surgical attention for these. An epidemiological study carried out in Uganda, demonstrated a 14% prevalence of untreated surgical conditions in children^6^. Many factors, including health care seeking, human resources and hospital capacity contribute to this situation, where service delivery does not meet the population’s need. The use of empirical findings to build an exploratory and theoretical frame for health-seeking and health-system barriers for children with hernia is important for designing strategies to improve access to and use of care.

Inguinal hernia occurs in 1–5% of all new-borns and between 9 and 11% of premature infants; it is the most common congenital abnormality among new-borns^7^. Surgery is the only effective treatment for groin hernia and it is recommended that all groin hernias in children should be operated as soon as possible. Umbilical hernias usually close spontaneously during the first years of life but may require surgery if this does not occur^8^. Elective hernia repair is a routine procedure in all health care systems and is relatively simple compared to other paediatric surgical procedures. For these reasons, hernia was chosen for this qualitative study. The study aimed to provide a better understanding among parents and health care providers of health care-seeking behaviour and the barriers to supplying adequate surgical care for children with hernia.

## Methods

### Study design

This was a qualitative, interview-based study.

### Definitions

A hernia is a protrusion of an organ through a defect in the structure normally containing it, and it is named according to the location where it occurs^9^. Health care-seeking behaviour (HSB) is defined as "any action or inaction undertaken by individuals who perceive themselves to have a health problem or to be ill, for the purpose of finding an appropriate remedy"^10^.

### Study context

The study was carried out in Soroti Regional Referral Hospital (SRRH) in eastern Uganda. A Regional Referral Hospital (RRH) is constructed to provide specialised services including surgery. A RRH has at least one employed general surgeon. It is a referral and support institution for lower levels hospitals and health units and also provides training for intern doctors, nurses etc., but not routinely for residents. SRRH is one of the 14 regional referral hospitals in Uganda, located in the Eastern region. It serves a predominantly rural population of 2.5 million people. SRRH has three employed consultant surgeons but no paediatric surgeon. There are only three paediatric surgeons in Uganda.

### Informants

The inclusion criteria for the parents were that their child must be below the age of 18 years at the time of the study and must have presented at Soroti Regional Referral Hospital with a hernia between November 2018 and February 2019. Children and their parents were recruited from the surgical outpatient clinic at Soroti Regional Referral Hospital. Exclusion criteria were unwillingness to participate or inability to give informed consent. All children received a hernia repair at the same hospital stay as when the interview was carried out.

Health care providers were recruited to the study by snowball sampling. Snowball sampling is a nonprobability sampling technique, where existing study subjects recruit future subjects from among their acquaintances. Thus, the sample group is said to grow like a rolling snowball^11^.

The aim of the recruitment process was to include enough informants to reach a high information power so that the aims of the study could be met^12^.

### Interviews

The parents were interviewed by the first author in a private room in the hospital and the interviews lasted 45 to 60 min. English or the local language, Ateso, was used depending on the informant’s preference. An in-depth semi-structured interview tool with ten open-ended questions was used (Appendix 1). The questions focused on knowledge and health care-seeking behaviour in order to identify barriers to seeking and receiving surgical health care.

The health care providers were interviewed in English by the first author using an interview guide (Appendix 2) to gather information about the management of inguinal hernia in children. The interviews lasted between 60 and 90 min.

All interviews were recorded with two digital voice recorders (Olympus) to ensure that no information was lost for technical reasons. All interviews were transcribed verbatim by the first author.

The first author translated the Ateso interviews and compared her translations with those by a professional translator. Whenever any differences in translation came up, the first author checked the translation, and consensus was reached.

### Data management

All data in the recorders was saved and locked in a safe place to which only the first author had the key. Copies of transcribed material was sent by Skype to the third author for reading, dialogue and validating with the first author. The data will be saved for 10 years according to Medical Research Council policy (MRC, June 2015).

### Qualitative thematic content analysis

The analysis was carried out using qualitative thematic content analysis as described by Burnard et al. This is a process starting from the transcribed text and step-by-step leading towards a higher level of condensation and abstraction^13^. The analysis was continuously carried out by the first author with the third author. The latter had access to the transcribed material and contributed to the precision of the content analysis by independently double coding as well as through discussions during the whole process. The first and third authors started open coding to create categories by reading independently the transcribed verbatim text, noting in the margin words, theories or short phrases that summed up what was being said in each interview. The second step was to match subcategories and group these into new categories. Meaningful units were identified consisting of paragraphs and phrases constituting a special meaning. The results were discussed every week on skype during data analysis by the first and third authors with continuous adjustments until these subcategories and categories were finally summarised into two main themes.

### Ethical considerations

This interview-based study carried no risk of physical harm and the clinical management of the children was not altered due to the study. The questions may cause a sense of unease, but this was not expressed by the respondents at the end of the interview when they were asked how they perceived the interview. Respondents were included after giving written or thumb-printed informed consent following oral and written information about the study. The informants received a transport refund from the first author. All data were coded and handled only by the authors. All data was de-identified prior to analysis.

The study was given ethical approval from Makerere University School of Public Health and the Uganda National Council of Science and Technology (Protocol Identity number is 542) and approved on April 22, 2018. The Hospital Director of SRRH gave written permission to conduct the study in the hospital. All methods were performed in accordance with the relevant guidelines and regulations.

## Results

**Figure 1:**
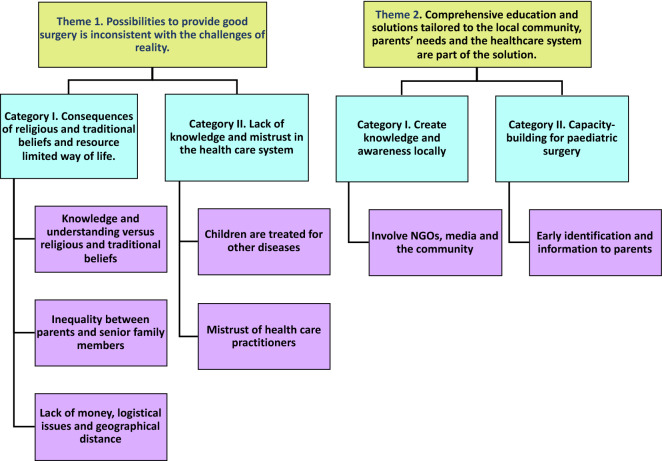
Themes, categories and sub-categories.

Twenty-six informants were included in this study: 14 parents with children with hernia (Table 1) and 12 health care providers (Table 2). The age of the children was between 1 and 6 years, most were boys and came from families of varying size (1–8 children per family). The parents were mostly mothers, and the ages ranged between 20 and 40 years. All but two parents were peasants. The health care workers were nurses, medical doctors, and clinical officers, most were women and the ages ranged between 26 and 46 years. During qualitative content analysis, seven sub-categories were identified and further condensed into four categories. These categories were organised into the following two themes: “*Possibilities to access and provide good surgical care are inconsistent with the challenges of reality*” and “*Comprehensive education and solutions tailored to local community, parents’ needs and the health care system are needed*” (Fig. 1).

**Table 1:** Basic characteristics of the children and their parents.

No	Child age (years)	Sex of child	Birth order	Diagnosis	Parentage (years)	Relation to child	Level of education of parent	Occupation of parent	Travel distance to Soroti RRH in km
1	2	F	6 out of 8	Umbilical hernia	38	Father	10 years in school	Peasant	50
2	2	M	4 out of 4	Groin hernia	22	Mother	6 years in school	Peasant	10
3	1	M	Only child	Groin hernia	20	Mother	11 years in school	Peasant	30
4	3	F	Only child	Umbilical hernia	23	Mother	7 years in school	Peasant	5
5	1.5	M	Only child	Groin hernia	24	Father	6 years in school	Peasant	20
6	1.5	M	4 out of 4	Groin hernia	30	Mother	6 years in school	Peasant	5
7	3.5	F	6 out of 8	Umbilical hernia	38	Father	10 years in school	Peasant	40
8	5.5	M	1 out of 2	Groin hernia	30	Mother	7 years in school	Peasant	10
9	1	M	Only child	Groin hernia	21	Mother	Tertiary Institution	Primary teacher	20
10	2	M	Only child	Groin hernia	20	Mother	5 years in school	Peasant	1.5
11	3	M	Only child	Groin hernia	24	Mother	8 years in school	Peasant	37
12	6	M	3 out of 6	Groin hernia	30	Mother	2 years in school	Peasant	50
13	4	M	7 out of 8	Groin hernia	40	Mother	3 years in school	Peasant	8
14	5	M	1 out of 2	Groin hernia	32	Mother	Tertiary institution	Primary teacher	7

**Table 2:** Basic characteristics of the health care providers.

No	Age (years)	Sex	Job title	Working experience (years)
1	41	Female	Nurse	3
2	31	Female	Medical doctor	2
3	38	Male	Nurse	9
4	26	Male	Nurse	1
5	40	Male	Clinical officer	9
6	32	Male	Nurse	3
7	31	Female	Medical doctor	2
8	37	Female	Medical doctor	4
9	28	Female	Medical doctor	2
10	39	Female	Nurse	15
11	39	Female	Clinical officer	5
12	46	Female	Nurse	20

## Theme 1. Possibilities to access and provide good surgery are inconsistent with the challenges of reality

### Category I. Consequences of religious and traditional beliefs and a resource limited way of life

#### Knowledge and understanding versus religious and traditional beliefs

Among conditions in children needing surgery, hernia was according to the health care providers, the most common, followed by accidents. The local name for hernia in the community is “simbibwa,” in lugishu, and in Ateso it is called “agulit.”

Most of the parents were aware that hernia is a common problem:

“I only know one condition; even children who were operated know that they have hernia. Some children have malaria, but now there are a number of children with hernia.”

“In my place we have hernia…and other problems with things inside the stomach.”

Several beliefs about the cause of hernia and its treatment were mentioned by the parents. Some believed that hernia was caused when the stump of the umbilicus failed to heal; or resulted from a stressful event:

“My child’s umbilicus did not heal well leaving an ulcer, I believe this is the cause of the hernia.”

“A disease that I got when I was pregnant, pre-eclampsia.”

Others believed that hernia was caused by witchcraft or sorcery and also talked about traditional beliefs such as cold winds. When a baby was born with a congenital abnormality or defect, the local community may blame the mother: *“It’s the mother’s fault”, or “She’s a witch” or “It’s the family planning she used that has caused this problem*”, or “*She’s useless for giving birth to an abnormal child”, or “This child is unlucky.”*

If a child was born with an obvious defect on exposed areas such as face, legs, or hands; some mothers hid it for fear of stigma, while fear of surgery or anaesthesia was a cause of delay in seeking surgical attention.

“I delayed bringing the child for surgery because one of the staff scared me. She told me that the drugs for anaesthesia were very strong so I should wait until he [son] is 6 years old.”

Some parents first used herbal remedies, and when these did not work, sought medical treatment. One parent said:

“There are local medicines for treating this hernia; my child was given this local medicine…... herbal remedy but no cure ‘Aaaa! I can’t manage this problem, and then I brought the child for surgery’.”

The parents felt powerless to seek medical help. They were worried about the sick child, and mothers were stressed. At the same time, they hoped that after surgery, the situation for the entire family would improve. A parent answered:

“If surgery is done, I would also go back home to look for school fees for the other children, and also take care of their needs.”

#### Inequality between parents and senior family members

Gender inequality manifested itself in diverse ways at household level. A common answer from the parents was that the sick child was a burden for the mother. Mothers were often the first parent willing to act but their influence and voice in decision-making concerning health care was often low and ignored. A health care provider remembered one case:

“The mother complained that when her child was sick, his father did not care about the problem.”

Old people are influential members of the family or community regarding a decision to seek health care. A parent said:

“I wanted to bring my child to the hospital, but this child’s grandmother had not okayed my decision, even if it was in the child’s best interest to get better.”

#### Lack of money, logistical issues and geographical distance

Lack of financial resources were important barriers to surgical care. The parents thought that their children needed surgery, because they did not recover after local treatment with herbs. Most parents wanted their children to be operated on as soon as possible but lacked money for hospital expenses. The following were frequent replies by the parents:

“This is the first time my child’s come to hospital, I delayed coming because I was told operation needed lots of money, come now because I heard there is a doctor who operates for free.” “I was desperate for money, due to costs incurred for surgery, I wanted my child operated on, however I had no money.” “Even in hospital I need money for food, and drugs.”

The long distance from home to hospital, and no money to pay for transport, were barriers for most of the children who were brought to hospital. A parent replied:

“It was a long distance to come hospital, and I didn’t have money for transport.”

Many of the mothers depended entirely on their husbands for permission to take the sick child to hospital, and for financial assistance. One informant described her situation:

“….. I did not have that money, which prompted me to take the child to Mukono (located several hours from Soroti Regional Referral Hospital. Researchers comment) where his father works, so that he could be treated from there……. took a child to health facility and was told to pay 200,000 Ug shillings. Ah! he [father]did not have that money, so I decided to return to the village.”

### Category II. Lack of knowledge and mistrust in the health care system

#### Children are treated for other diseases

Some of the health care providers observed that in lower-level health units, or private clinics, many children presenting with vomiting were treated as malaria, but some of these children could be having intestinal obstructions due to obstructed hernia. This caused delays, so by the time a child was brought to hospital he/she was very weak. The health care providers stated that when they ask parents why they have not brought their children for treatment earlier; the following were common answers:

“The health care worker who saw my child in the private clinic told me he had malaria, and has been giving him antimalarial drugs, but when his condition got worse, I was told to come to hospital where the hernia was diagnosed.” “A parent had brought her child for treatment because he had malaria, but after examining him, the doctor told her that he also had hernia.”

At the time of hospitalization and surgery, the parents requested more information about how to care for the operated child:

“Will I be required to buy drugs for my child after surgery?”

“Once my child is operated on, can I bring him for wound dressing? Any special diet, and should water touch the operation site or dress it?”

#### Mistrust of health care providers

A parent brought her boy with hernia to hospital, where he was admitted but not operated on. He was re-scheduled five times, and then the mother lamented, it could have been due to lack of qualified staff. One parent replied:

“I prayed to God to intervene so that the doctors would operate on my child and heal him.”

One parent did not trust the doctor’s hernia diagnosis, which resulted in unnecessary investigations and expenditure, before the child came to hospital for surgery. Some informants blamed the health care providers for delay of treatment. One parent said:

“My child was admitted to hospital many times, but the hernia diagnosis was missed, other co-existing conditions were treated.”

Being a government facility, the hospital offers free services; but some staff extorted money from patients. Often these people remained unknown. One parent said:

“My money was taken by one of the hospital staff.”

One interview with a parent ran as follows:

Parent: *“Someone asked me for money for surgery.”*

Interviewer: *“Can you tell me their name?”*

Parent: *“Money was not taken from me, but my sister is the one who gave the money.”*

Interviewer: *“Please tell her to come.”*

Parent: *“Sorry she has already gone home and is not coming back.”*

## Theme 2: Comprehensive education and solutions tailored to the local community, parents’ needs and the healthcare system are part of the solution

### Category I. Create knowledge and awareness locally

#### Involve NGOs, media and the community

The health care providers said that health services for vulnerable people such as children should be cost-neutral. Collaboration with an NGO can help trace children with surgical conditions in the community and refer them to hospital for surgery. A health care provider gave examples:

“NGOs such as Compassion Africa and World Vision have been bringing children with surgical needs to hospital, and sometimes pay for their needs (drugs, food).”

The lessons learnt among the parents who came to the hospital were that surgery is needed for a child with hernia. That would also reduce the parents’ economic burden. One parent stated that she would like to inform her community about this on return.

“Once I get back home, after my child’s surgery; I will inform the community that the only treatment for hernia is surgery and not herbal remedy.”

It was recommended that health care providers should organize radio talk shows, involve local leaders in dissemination of information about hernia at community gatherings, thus creating awareness including that surgical conditions like hernia are managed by surgery. A health care provider gave a concrete example:

“You can get a topic like hernia and educate the community about it on the radio.”

Health care providers also need to advise traditional healers to send children with surgical conditions to the hospital and encourage the parents to bring these children for surgery. A health care provider gave the following advice:

“….as a health care provider, it is important to have discussions with the traditional healers and give them advice because the community has so much trust in them. Whenever a child is sick, the traditional healers are visited first, causing delays…”

### Category II. Capacity-building for paediatric surgery

#### Early identification and information to parents

The parents requested trained doctors to provide health care services for the children. Two parents proposed:

“You people with knowledge should help give talks to the community about hernia, because parents undergo a lot of stress when they realize that a child is sick.”

“You people who are trained should be willing to help us.”

Most hospitals in Uganda lack intensive care units (ICUs) and equipment for children, no constant supply of blood, and yet children need specialized management. Lack of these can cost lives. Two health care providers said:

“For example, this hospital does not have an ICU, and often surgery is postponed due to lack of blood.” “As we do not have an ICU, or specialized team to manage children who are very sick, some die.”

Lack of hospital staff meant that postoperative monitoring of the children was difficult. A health care provider expressed that:

“You can find one general nurse managing both children and adults in a general ward; we really need a paediatric ward and paediatric nurses.”

There was a wish among the health care providers to build a surgical team consisting of surgeons, paediatric doctors, paediatric nurses and anaesthesiologists to manage sick children. They stated that there is a demand for more knowledge in order to manage stressed parents who have children with surgical health problems.

“Request for capacity-building of surgical team (nurses, anaesthetists, doctors), equip them with knowledge and skills so they can meet the community’s paediatric surgical needs.”

Early identification of surgical conditions was desired. Ultrasound as part of antenatal care and thorough physical examination at birth, plus information, including a plan, to parents was recommended. A health care provider gave the following example:

“If a baby is born with abnormality such as hernia, the mother is informed, and the advice of a surgeon sought.”

## Discussion

This study found that parents had sought health care on several occasions before the children could finally undergo surgery for hernia. Barriers to surgical care related both to lack of awareness and knowledge among parents; but also, among health care providers, as hernias were sometimes missed, and children received treatment for other conditions instead. From the health care worker’s perspective, the intention to provide surgical care of high quality conflicted with the ability to do so in reality. Structural limitations such as the lack of an intensive care unit, adequate perioperative care and the lack of a team dedicated to the management of children with surgical conditions were identified as limiting factors for the quality of care.

Children depend on their caregivers for their health and wellbeing and caregivers’ adequate health-seeking behaviour may help reduce morbidity and mortality^14–16^. Qualitative research relating to child surgery is sparse but exists for other common paediatric conditions. A systematic review of the recognition of disease and care-seeking for three common childhood illnesses in low and middle-income countries (LMICs) was not enough^17^. Another systematic review, of caregivers’ treatment-seeking behaviour and practice in Uganda concluded that home management remains a usual first response for malaria, with most caregivers seeking treatment if the child does not improve^15^. Similarly, the present findings confirmed that children with hernia are first given treatment at home in the local community setting before being brought to hospital. Inadequate knowledge combined with religious and traditional beliefs are barriers identified in this study, and these contribute to the delay in seeking care.

Surgery is the only effective treatment for hernia, and increased community awareness and knowledge of this common condition could potentially reduce the first delay in seeking health care and may reduce expenditure related to traditional medicine. Community information strategies including radio, local leadership and patient representatives, and targeted information to pregnant women in hospital, were suggested by both parents and health care providers.

Gender inequality in the community and in the families was an important concern that limited access to surgery for children. A previous study investigated the association between women’s’ autonomy and gender norms in relation to maternal and child health outcomes in eight African countries, including Uganda. Women with a high level of autonomy were more likely to bring children with an acute respiratory infection (ARI) to a health care facility than were women with low autonomy. Women with positive gender norms were more likely to have fully immunized children, compared to women without^18^. Hernia has in common with ARIs and immunisation that access to a health care facility with trained staff is necessary for its successful treatment. Therefore, education and empowerment of women is likely to affect children’s access to surgical services.

Lack of financial resources was identified as a barrier to seek and receive surgical care. The present results are unsurprising and have been documented in previous research assessing barriers to surgical care in general and for children in Uganda in particular^6,19^. Financial protection, also for indirect costs, will be necessary and collaboration with NGOs for paediatric surgery were suggested as part of the solution. Transparency must be improved to avoid extortion of money from parents.

Community involvement was suggested by both parents and health care providers and should be prioritised in future efforts for paediatric surgery. For interventions to become successful, it is important that they are created and implemented by academia together with both providers and end-users^20^. Community engagement can lead to improved health and health behaviour among disadvantaged populations through effective community consultation and participation^21^.

The organisation of a surgical team to manage children with surgical conditions was proposed and this team should include nurses, surgeons, paediatric physicians and paediatric anaesthesiologists. Such a team would also be in charge of the development and maintenance of surgical services. An additional task for this team could be to organise and follow up awareness programmes in the community as well as to inform health care workers at lower-level health units about paediatric surgical conditions. This has potential to improve the detection rate of surgical conditions in children and subsequent referral to an appropriate hospital.

Qualitative research is evaluated in relation to its trustworthiness. Trustworthiness has three central aspects: credibility, dependability and transferability^22^. Credibility is high as semi-structured interviews and thematic content analysis form a suitable method for exploring attitudes, experience, and perceptions. The first and third authors did independent coding of the interviews. Further, each respondent evaluated the interview as positive in the end of the interview. Dependability is good as the data were collected during a short period, minimising the risk due to uncontrollable external factors influencing changes in the material and analysis over time. Transferability refers to whether the findings can be transferred to other groups and contexts. This study was carried out in one hospital only, where the special conditions at that hospital may have influenced their perception, reducing transferability. However, delayed access to surgical health care for children is a problem all over Uganda and it is plausible that similar reasons obtain in other hospitals too.

The informants’ answers may have been influenced by the fact that they were interviewed by a surgeon, the first author. On the other hand, the interviewer was fluent in the local language and also grew up in the study setting, which probably had a positive impact on the trust between interviewer and study participants.

## Conclusion

Strategies to promote access to surgical services for children with hernia and other surgical conditions should focus both on the local community setting in which the children and their parents live and the health care facilities and system. Local community involvement is desired. A package that includes information dissemination and knowledge improvement, the formation of a team that can tailor surgical care for children in the individual facility and ways to prevent financial extorsion has the potential to increase access and quality of surgical services for children.

## Supplementary Information

Supplementary Information 1.

Supplementary Information 2.
