# Supplementary material for: Barriers and potential solutions for improved surgical care for children with hernia in Eastern Uganda
Source: Sci Rep. 2021 May 31;11:11344. doi: 10.1038/s41598-021-90717-2 (PMC8166922; doi:10.1038/s41598-021-90717-2)
Supplement: Supplementary file 1 — Supplementary Information 1. [file 41598_2021_90717_MOESM1_ESM.docx]

# CARE GIVERS IN-DEPTH INTERVIEW GUIDE: of surgical conditions in children, and health seeking behaviour of their parents/guardians in Eastern Uganda- a cross sectional study in the Iganga and Mayuge Districts Health and Demographic Surveillance Site

***(Note Interviewer: Child with will be present with the carer, either at home/in clinic, or at the hospital.***

**Name of Interviewer________________________**

**Date____________________________________**

**Name of Interviewee_______________________**

**Residence 🞏 Urban__ 🞏 Rural**

**Sex____________________________________**

**Age____________________________________**

**Education level________________________**

**Marital status________________________**

**Place of Interview _______________________**

**Time ____________________________**

1: Could you please share with us the common health conditions in children in your community?

2.I'd like to start by having you briefly tell me about your child (child identified to have the condition) and how you got to know that he/she has this condition. *(Note to interviewer: You may need to probe to gather the information you need, age of child, where the child was delivered from when/where diagnosis was made, by whom, how long child has had this condition)*.

2. Can you tell me what happened when you first sought help for your child? (Note to interviewer: Where child was taken first, reasons why, what exactly did she experience?)

3. How are you now managing this child? (Note to interviewer: *If so, probe - “Any special place child is taken to?” “What facilities if any are available?”, “What is done?” Any local or western medicines received?” “If so from where, and name of the treatments”)*

4."Are you aware of any local terminologies used to describe a child like this one?" *(Note to interviewer: If so, probe - "What are the terms used?”,” What do these terms mean?”, "Do you know why these terms are used?”)*

5. What do you think is the cause of your child’s condition?  *(Note to interviewer: probe –for as many causes as possible to gather the information that you need.” Can you explain……”)*

6. What concerns have you had or do you have regarding the management of this child? *(Note to interviewer: You may need to probe to gather the information you need-concerns in the health unit, at home, in the community, etc)*

7. What other problems/challenges are you experiencing? Can you share your most challenging experience? *(Note to interviewer: You may need to probe to gather the information you need)*

8. What is the main aspiration you have regarding the management of your child? *(Note to interviewer: probe- “the main concern that the caregiver would wish to be realised in her/his child should be indicted here)*

9. Is there any other information about the children like this in this area that you think would be useful for me to know? *(Note to interviewer: If so, you may need to probe to gather the information you need)*

10.Is there anything more you would like to add?

*I’ll be analysing the information you and others have given me. I hope to submit a draft report to the district in one month’s time. I’ll be happy to send you a copy to review at that time, if you are interested*.

**Thank you for your time.**
