# Supplementary material for: Barriers and potential solutions for improved surgical care for children with hernia in Eastern Uganda
Source: Sci Rep. 2021 May 31;11:11344. doi: 10.1038/s41598-021-90717-2 (PMC8166922; doi:10.1038/s41598-021-90717-2)
Supplement: Supplementary file 2 — Supplementary Information 2. [file 41598_2021_90717_MOESM2_ESM.docx]

**APPENDIX 3:**

# HEALTH SERVICE PROVIDERS KEY INFORMANT GUIDE ON

**Health seeking behaviour for groin hernia in children in Eastern Uganda-identifying barriers to care and their solutions.**

**Name of Interviewer________________________**

**Date____________________________________**

**Staff Position____________________________**

**Sex____________________________________**

**Age____________________________________**

**Name of Health Unit________________________**

**Place of Interview _______________________**

**Time ____________________________**

1. I’d like to start by having you briefly describe your responsibilities and involvement thus far with this Health Unit/Hospital. *(Note to interviewer: Probe for how long the provider has been there, you may need to probe to gather the information you need)*.

2. Can you share with me some of the paediatric common surgical conditions you know (can you share with me the ones you know in this community?)

3. In your opinion do you think these paediatric surgical conditions are a problem? To what extent are they in Soroti Regional Referral hospital.

5. What is your professional experience with managing children with hernia requiring intervention by surgery? (Note to interviewer: If so, probe - “Any training these conditions?” “Where trained?” “When trained? Number of children seen in a month with a surgical condition”?)

**"I'm now going to ask you some questions that I would like you to answer to the best of your ability. If you do not know the answer, please say so."**

**Give Key informant a description of paediatric surgical condition you know, and how you have been managing them in this health facility**

6."Are you aware of any local terminologies used to describe such a child with hernia give examples?" *(Note to interviewer: If so, probe – Do they affect health seeking behaviour in this community? "What are the terms used?” What do these terms mean?”, "Do you know why these terms are used?”)*

7. When parents/caregivers have a child with hernia what do they do to seek care? (Note to interviewer: You may need to probe to gather the information you need. (First point of contact, reasons why?);

8. Is there any other information about the children with surgical conditions in this area that you think would be useful for me to know? *(Note to interviewer: If so, you may need to probe to gather the information you need*

9. What recommendations do you have for the future management children with hernia

10. Is there anything more you would like to add?

*I’ll be analysing the information you and others have given me, I’ll be happy to send you a copy to review, if you are interested.*

**Thank you for your time.**
